# Milrinone for the treatment of heart failure caused by severe Pneumonia in children with congenital heart disease: a meta-analysis

**DOI:** 10.1186/s12887-023-04360-z

**Published:** 2023-10-28

**Authors:** Wenshen Shao, Shuangshuang Diao, Lu Zhou, Lina Cai

**Affiliations:** Department of Cardiothoracic Surgery, Children’s Hospital of Nanjing Medical University, Nanjing, China

**Keywords:** Milrinone, Congenital Heart Disease, Children, Pneumonia, Heart Failure, Meta-analysis, Treatment, Care

## Abstract

**Background:**

Children with congenital heart disease (CHD) are easily complicated by severe pneumonia and heart failure. We aimed to conduct a meta-analysis to evaluate the effects and safety of milrinone for the treatment of heart failure caused by severe pneumonia in children with CHD to provide evidence for the clinical CHD treatment.

**Methods:**

Two authors searched MEDLINE, PubMed, Embase, Science Direct, Cochrane Central Register of Controlled Trials, the Cochrane Library, Wanfang database, Chinese Biomedical Literature Database, China National Knowledge Infrastructure (CNKI) for randomized controlled trials (RCTs) about the application of milrinone in the treatment of heart failure caused by severe pneumonia in children with CHD in children up to December 10, 2022. Two evaluators independently selected the literature, extracted data and evaluated the methodological quality, meta-analysis was carried out with RevMan 5.3 software.

**Results:**

Eight RCTs involving 680 CHD children complicated by severe pneumonia and heart failure were included in this meta-analysis. Meta-analysis indicated that total effective rate of the milrinone group was higher than that of control group (RR = 1.25, 95%CI: 1.17 ~ 1.34, P < 0.001), the time to stable heart rate of the milrinone group was less than that of control group (RR=-0.88, 95%CI: -1.09~ -0.67, P < 0.001). The time to stable respiration of the milrinone group was less than that of control group (RR=-0.98, 95%CI: -1.17~ -0.78, P < 0.001). The LVEF of the milrinone group was higher than that of control group (RR = 6.46, 95%CI: 5.30 ~ 7.62, P < 0.001). There was no significant difference in the incidence of adverse reactions between the milrinone group and control group (RR = 0.85, 95%CI: 0.47 ~ 1.56, P = 0.061). Funnel plots and Egger regression test results indicated that there were no statistical publication bias amongst the synthesized outcomes (all P > 0.05).

**Conclusions:**

Milrinone is beneficial to improve clinical symptoms and cardiac function and increase the therapeutic effect and safety in children with CHD complicated by severe pneumonia and heart failure. However, more RCTs with large samples and rigorous design are needed to verify this finding.

## Background

Congenital heart disease (CHD) is a common type of heart disease in pediatrics, with an incidence of 9% ~ 13%, which poses a serious threat to the health and safety of children [1]. CHD children are often complicated by severe pneumonia associated with acidosis, inflammation, hypoxia blood circulation disorders et al. The increased pulmonary artery pressure, coupled with cardiac myocyte damage and special intracardiac structure, making CHD children prone to heart failure [2]. Children with CHD complicated by severe pneumonia and heart failure are ofen in critical condition without typical symptoms in the early stage, so it is easy to miss the best time for treatment [3, 4]. Therefore, for children with CHD complicated by severe pneumonia and heart failure, the etiology should be confirmed in time, the cardiac load should be relieved, the inflammatory reaction should be reduced, and the recovery of myocardial function should be promoted [5, 6]. Therefore, exploring safe and effective treatments are very important for CHD patients complicated by severe pneumonia and heart failure.

Milrinone is a phosphodiesterase inhibitor of the second generation, which can be used in cardiac support therapy in patients with acute heart failure, pulmonary hypertension or chronic heart failure [7, 8]. In recent years, there are many reports about the use of milrinone injection combined with routine methods in the treatment of severe pneumonia and heart failure in children with CHD, but the sample size is small, and the results are inconsistent [9]. Therefore, the purpose of this study is to explore the efficacy and safety of milrinone in the treatment of heart failure caused by severe pneumonia in children with CHD by means of meta-analysis and systematic review, to provide evidence-based medicine reference for clinical treatment and nursing care of CHD.

## Methods

This meta-analysis was conducted and reported according to the Preferred Reporting Items for Systematic reviews and Meta-Analyses (PRISMA) statement [10].

### Literature search and retrieval

Two authors searched MEDLINE, PubMed, Embase, Science Direct, Cochrane Central Register of Controlled Trials, the Cochrane Library, Wanfang database, Chinese Biomedical Literature Database, China National Knowledge Infrastructure (CNKI) databases. We searched for the randomized controlled trials (RCTs) about the application of milrinone in the treatment of heart failure caused by severe pneumonia in children with CHD. The references included in the literature were followed up, and the retrieval time limit was from the establishment of the database to December 10, 2022. The literature search strategy was: (“milrinone”) AND (“children” OR “child” OR “pediatric”) AND (“congenital heart disease” OR “severe pneumonia” OR “heart failure”) AND (“randomized controlled trial” OR “RCT”). In this study, the method of combining free words with subject words was used for database retrieval.

### Inclusion and exclusion criteria

The inclusion criteria of this meta-analysis were as follows: (1) the age of the children was 0–18 years old; (2) the RCT of children with CHD complicated by severe pneumonia and heart failure treated with milrinone injection, in which the control group was given routine treatment, and the milrinone group was given routine symptomatic treatment plus milrinone treatment; (3) the corresponding outcome indicators, such as effective rate and adverse reactions, were reported in the literature. The exclusion criteria of this meta-analysis were: (1) literature reports with incomplete data; (2) repeatedly published literature.

### Data collection

Two researchers read the titles and abstracts of the literature independently. After excluding the literature that obviously did not conform to the inclusion criteria, they completed the preliminary screening of the full text of RCT which met the inclusion criteria, checked the results of inclusion in RCT with each other, and evaluated the quality of methodology. If there were any differences, the third researcher resolved disagreement after discission on whether the report met the relevant inclusion and exclusion criteria. We collected the following data from the included RCTs: the first author and country; publication time; general data of the milrinone group and control group; intervention measures; efficacy indicators and adverse reactions. If the clinical test data were incomplete, we contacted the original author as much as possible.

The main results collected were as follows: the total effective rate (total effective rate = number of effective cases / total number of cases treated × 100%), the time to stable heart rate; the time to stable respiration; left ventricular ejection fraction (LVEF); the adverse reactions: arrhythmia, hypotension, fatigue, nausea, vomiting, diarrhea, allergy.

### Quality evaluation of included RCTs

The quality of the included RCTs and the risk of bias of each study were assessed according to the Cochrane Collaboration recommendations of the Cochrane handbook of systematic reviews of interventions, which included following items: sequence generation, allocation sequence concealment, blinding, incomplete outcome data, selective outcome reporting, and other sources of bias. Quality evaluation of the risk of bias were conducted independently by two investigators. Disagreements were resolved by further discussion until consensus was reached.

### Statistical method

We used Rev Man 5.3 software for meta-analysis, the measurement data were evaluated by mean difference (MD) and 95% confidence interval(95%CI), and the binary or multi-classified data were evaluated by relative risk (RR) and 95%CI. I^2^ test was used to judge heterogeneity, I^2^ > 50% showed that the heterogeneity difference was statistically significant, random effect model was used for meta-analysis. I^2^ < 50% showed that heterogeneity difference had no statistical difference, and fixed effect model was used for meta-analysis. Funnel plot and Egger regression test was used to identify publication bias. Sensitivity analyses were conducted for each synthesized outcomes to identify possible sources of heterogeneity. The difference between groups was considered to be statistically significant when P < 0. 05.

## Results

**Fig. 1:**
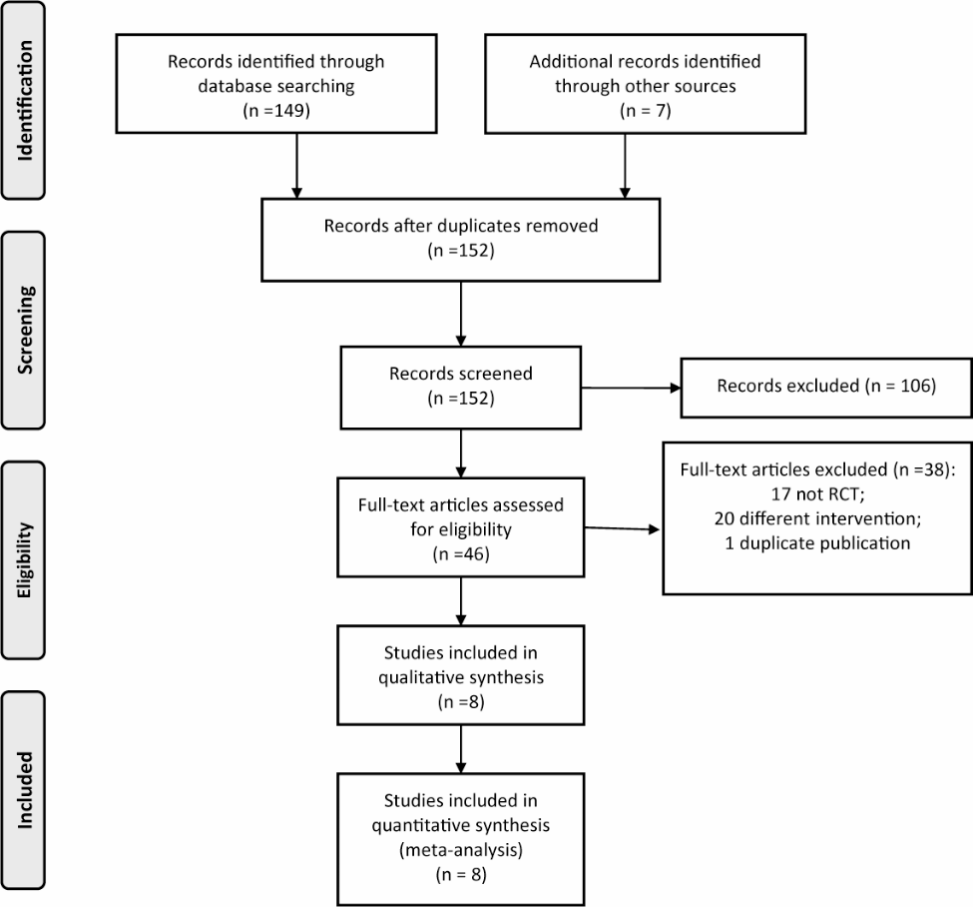
PRISMA flow diagram of study selection

At the beginning, a total of 156 articles were retrieved. After reading the title, abstract and full text based on the inclusion and exclusion criteria, eight RCTs [11–18] were included in this meta-analysis. A total of 680 CHD children complicated by severe pneumonia and heart failure were involved, including 341 cases in the milrinone group and 339 cases in the control group. The flow chart of literature screening is shown in Fig. 1, and the basic characteristics of the literature are shown in Table 1.

**Table 1:** The characteristics of included RCTs

RCT	Sample size			Age			Interventions		Outcomes
	Milrinone group	Control group		Milrinone group	Control group		Milrinone group	Control group	
Chen 2015	25	25		1.7 ± 0.5	1.5 ± 0.3		Routine treatment + milrinone injection intravenous drip at 0.5 pg/µg/(kg-min).	Routine treatment	①⑤
Huang 2017	55	55		1.78 ± 0.41	1.74 ± 0.42		Routine treatment + milrinone injection intravenous drip at 0.5 pg/µg/(kg-min).	Routine treatment	①②③⑤
Lin 2021	48	46		4.5 ± 0.7	4.7 ± 0.8		Routine treatment + milrinone injection intravenous drip at 0.25 ~ 1 µg/(kg-min).	Routine treatment	①⑤
Ma 2020	39	39		2.84 ± 0.43	2.91 ± 0.46		Routine treatment + milrinone injection intravenous drip at 0.05 µg/(kg-min).	Routine treatment	①②③⑤
Tang 2018	43	43		1.4 ± 0.3	1.3 ± 0.2		Routine treatment + milrinone injection intravenous drip at 0.25 ~ 0.75 µg/(kg-min).	Routine treatment	①②③④⑤
Wang 2020	31	31		0.41 ± 0.02	0.40 ± 0.02		Routine treatment + milrinone injection intravenous drip at 0.5 pg/µg/(kg-min).	Routine treatment	①⑤
Wu 2016	50	50		7.28 ± 1.39	7.2 ± 1.3		Routine treatment + milrinone injection intravenous drip at 0.25 ~ 0.75 µg/(kg-min).	Routine treatment	①④⑤
Wu 2017	50	50		7.28 ± 1.39	7. 2 ± 1. 3		Routine treatment + milrinone injection intravenous drip at 0.25 ~ 0.75 µg/(kg-min).	Routine treatment	①⑤

Notes: RCT, randomized controlled trial; ①, the total effective rate; ②, the time to stable heart rate; ③, the time to stable respiration; ④, LVEF; ⑤, the incidence of adverse reactions

**Fig. 2:**
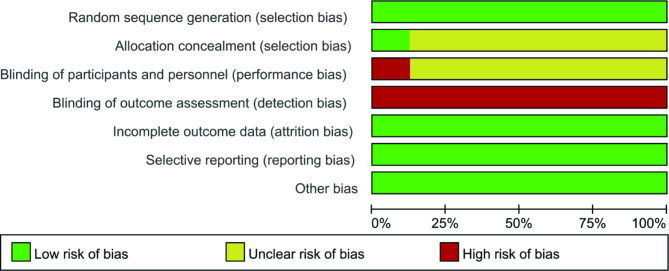
Risk of bias graph

**Fig. 3:**
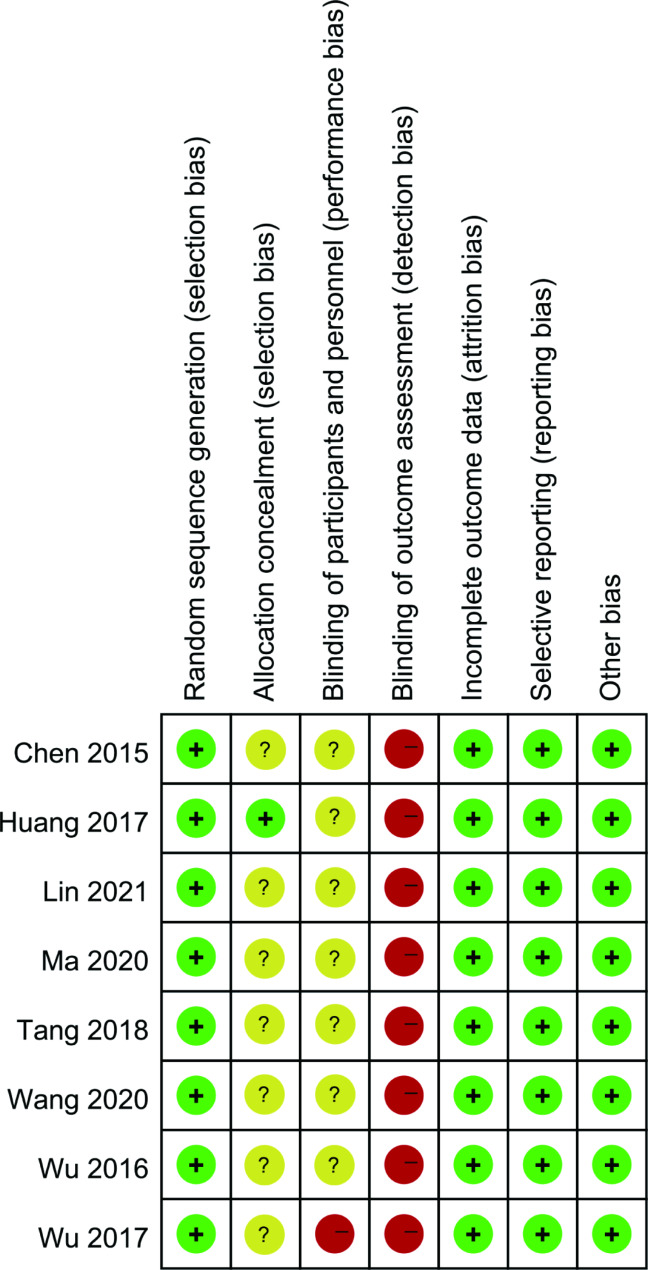
Risk of bias summary

The quality results of the study included in RCTs are shown in Figs. 2 and 3. The overall quality of the research included in RCTs was good. All the included RCTs [11–18] reported the methods of randomized sequence generation. Most included RCTs did not report the allocation concealment and blind design. No other risk of bias was found. Among the eight articles included, the main type of CHD was left-to-right shunt, and there was no specific difference in the CHD types between the milrinone group and the control group.

**Fig. 4:**
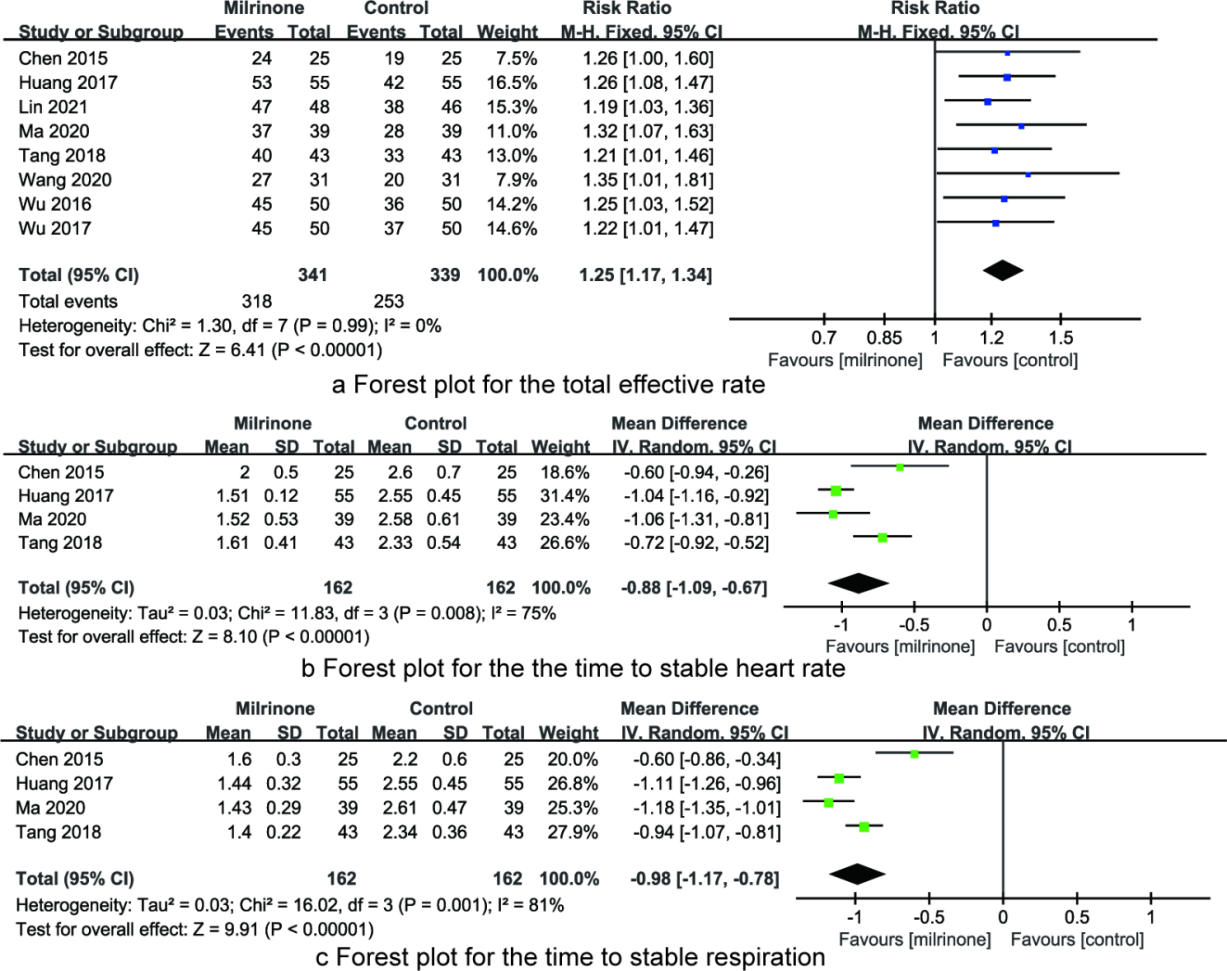
The forest plots for synthesized outcomes (**a**. total effective rate; **b**. time to stable heart rate; **c**. time to stable respiration.)

*The total effective rate* A total of eight articles reported the total effective rate, including 341 CHD children in the milrinone group and 339 CHD children in the control group. Since there was no heterogeneity on the total effective rate among the studies (I^2^ = 0%, P = 0. 99), the fixed effect model was used for meta-analysis. The results showed that the total effective rate of the milrinone group was higher than that of control group (RR = 1.25, 95%CI: 1.17 ~ 1.34, P < 0.001, Fig. 4a).

*The time to stable heart rate* A total of four articles reported the time to stable heart rate, including 162 CHD children in the milrinone group and 162 CHD children in the control group. Since there was heterogeneity on the time to stable heart rate among the studies (I^2^ = 75%, P = 0. 008), the random effect model was used for meta-analysis. The results showed that the time to stable heart rate of the milrinone group was less than that of control group (RR=-0.88, 95%CI: -1.09~ -0.67, P < 0.001, Fig. 4b).

*The time to stable respiration* A total of four articles reported the time to stable respiration, including 162 CHD children in the milrinone group and 162 CHD children in the control group. There was heterogeneity on the time to stable respiration among the studies (I^2^ = 81%, P = 0. 001), so the random effect model was used for meta-analysis. The results showed that the time to stable respiration of the milrinone group was less than that of control group (RR=-0.98, 95%CI: -1.17~ -0.78, P < 0.001, Fig. 4c).

Figure 4 The forest plots for synthesized outcomes (a. total effective rate; b. time to stable heart rate; c. time to stable respiration.)

**Fig. 5:**
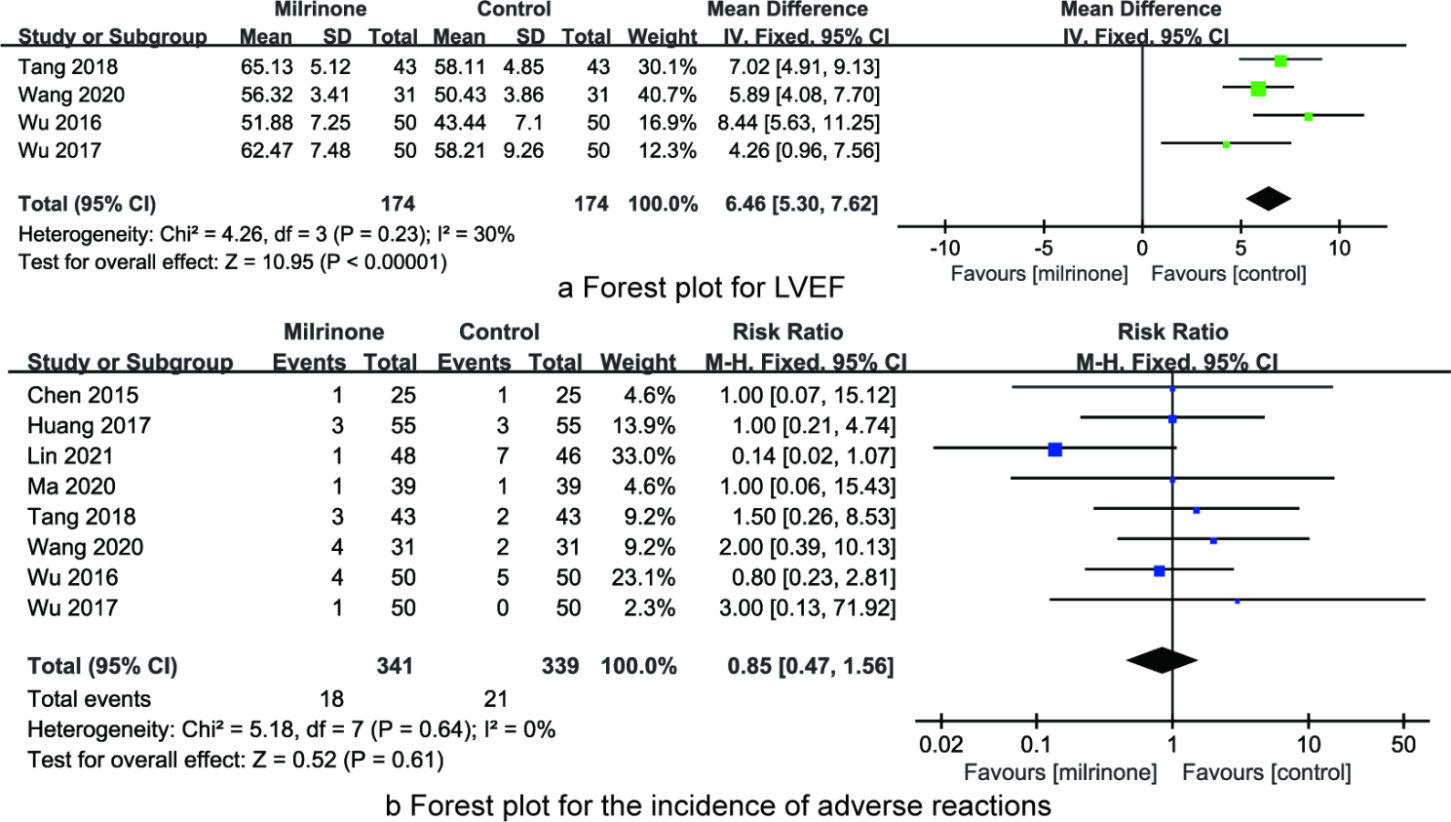
The forest plots for synthesized outcomes (**a**, LVEF; **b**, incidence of adverse reactions.)

*LVEF* A total of four articles reported the LVEF, including 174 CHD children in the milrinone group and 174 CHD children in the control group. There was no heterogeneity on the LVEF among the studies (I^2^ = 30%, P = 0. 23), so the fixed effect model was used for meta-analysis. The results showed that the LVEF of the milrinone group was higher than that of control group (RR = 6.46, 95%CI: 5.30 ~ 7.62, P < 0.001, Fig. 5a).

*The incidence of adverse reactions* A total of eight articles reported the incidence of adverse reactions, including 341 CHD children in the milrinone group and 339 CHD children in the control group. Since there was no heterogeneity on the incidence of adverse reactions among the studies (I^2^ = 0%, P = 0. 64), the fixed effect model was used for meta-analysis. And the results showed that there was no significant difference in the incidence of adverse reactions between the milrinone group and control group (RR = 0.85, 95%CI: 0.47 ~ 1.56, P = 0.061, Fig. 5b).

**Fig. 6:**
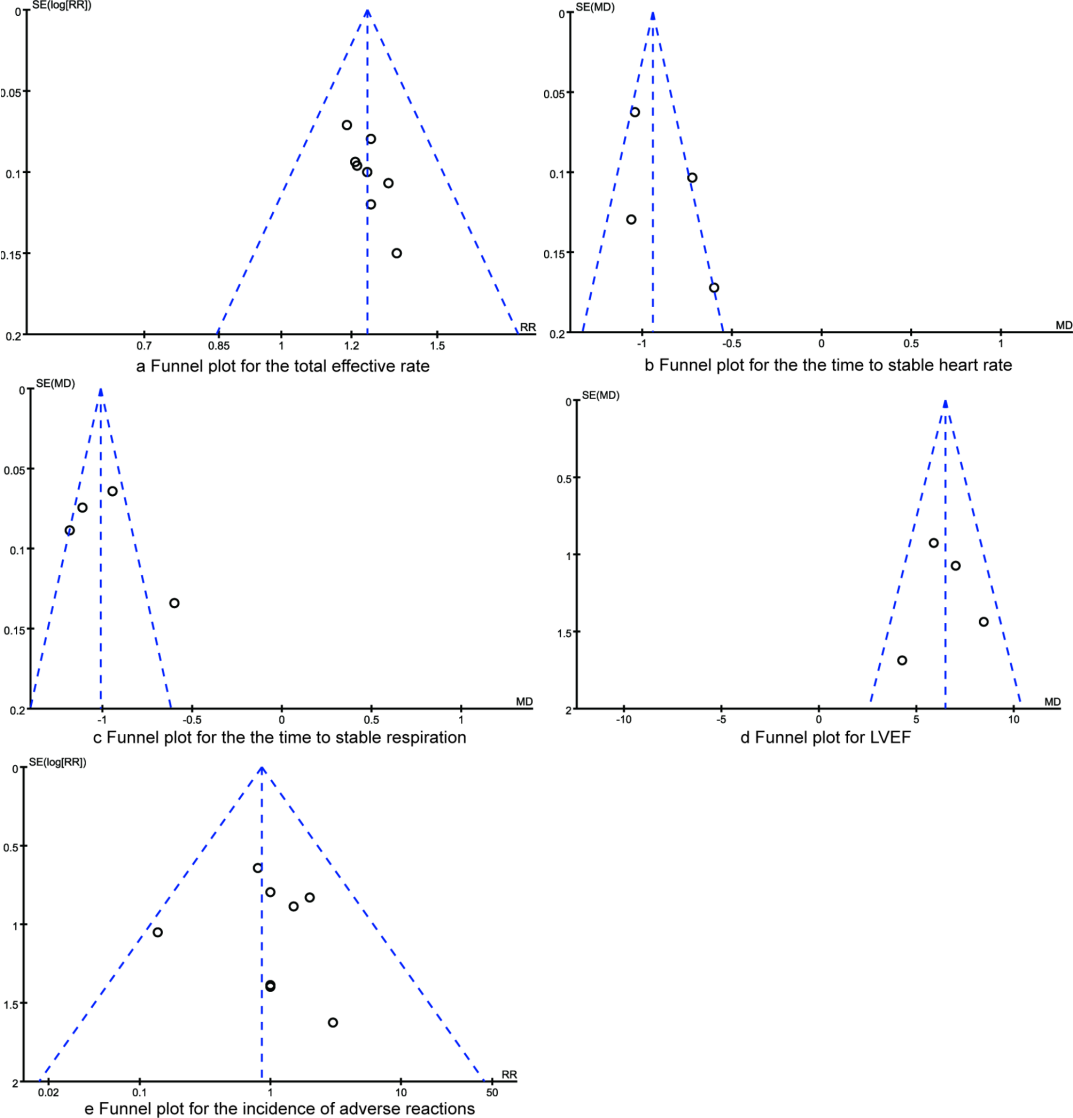
The funnel plots for synthesized outcomes (**a**. total effective rate; **b**. time to stable heart rate; **c**. time to stable respiration, **d**. LVEF; **e**. incidence of adverse reactions.)

The publication bias analysis of the results of each study is shown in Fig. 6. As shown in the funnel plots, the included studies were basically symmetrical on both sides of the funnel plots. Egger regression test results indicated that there were no statistical publication bias amongst the synthesized outcomes (all P > 0.05).

After excluding RCT one by one on every analyzed result to check that if the overall results changed accordingly, we found that the overall results were not changed by excluding any included RCTs, indicating that these results had good stability.

## Discussions

CHD is a common congenital malformation in pediatric patients. CHD children are often complicated by pulmonary congestion, poor immunity and high incidence of respiratory tract infection, which delay the growth and development of CHD children [19]. Children easily complicate myocardial damage during the natural course and treatment of CHD [20]. Once pneumonia occurs repeatedly or is aggravated, it can lead to heart failure, which is difficult to treat. At present, for the treatment of CHD complicated by severe pneumonia and heart failure, cardiotonic therapy are taken as the fundamental measures, and anti-infection, oxygen inhalation and diuresis are taken as assistant measures to improve the cardiac function of children [21, 22]. Digitalis is a commonly used anti-heart failure drug. By inhibiting Na-K-ATP enzyme in myocardial cell membrane, digitalis increases the level of Na + in fine cells, in turn promotes the exchange of Na + and Ca 2 +, increases the level of intracellular Ca 2 +, exerts positive inotropic effect, reduces the activity of sympathetic nervous system and renin-angiotensin, and restores the inhibitory effect of baroreceptor on sympathetic impulses from the central nervous system [23, 24]. However, digitalis takes effect slowly, and it may increase myocardial oxygen consumption. Besides, the therapeutic dose of digitalis is close to the toxic dose, so the patients is prone to have toxic reactions such as arrhythmia and gastrointestinal discomfort [25, 26]. Milrinone is a phosphodiesterase inhibitor of the second generation, which can be used for cardiac support in patients with acute heart failure, pulmonary hypertension or chronic heart failure [27]. Milrinone plays a role in increasing cardiac output, improving left ventricle-artery coupling and improving cardiac mechanical efficiency by improving myocardial contractility, cardiac relaxation and inducing vasodilation [28]. Milrinone has relatively few adverse reactions, however some patients may have headache, tachycardia, decreased platelet count and so on. Related studies have shown that milrinone can reduce the risk of low cardiac output syndrome after CHD in children, but it is also an independent risk factor for tachyarrhythmia after CHD [29, 30].

Milrinone is a new generation of positive inotropic drug, which can selectively inhibit phosphodiesterase III in cardiomyocytes, increase the content of cyclic adenosine monophosphate and increase the concentration of calcium in the cytoplasm of cardiomyocytes [31, 32]. In addition, milrinone can increase the content of cyclic adenosine monophosphate in vascular smooth muscle, reduce the release of calcium from sarcoplasmic reticulum, thereby increases the concentration of calcium in sarcoplasmic reticulum, improves ventricular diastolic compliance, and does not increase myocardial oxygen consumption and affect heart rate [33–35]. The results of this meta-analysis have shown that compared with the control group, milrinone have obvious advantages in total effective rate, time to stable heart rate, time to stable respiration and improvement of LVEF. There is no significant difference in the incidence of adverse reaction between milrinone group and control group, indicating that routine treatment combined with milrinone in the treatment of CHD complicated by severe pneumonia and heart failure has good effects and safety, it can quickly improve the symptoms and signs of children, promote the recovery of cardiac function.

In the treatment of children with congenital heart disease complicated by acute heart failure, the advantage of milrinone is that it can significantly improve the cardiac function of children [36]. Compared with children with simple heart failure, the treatment of congenital heart disease with acute heart failure is more difficult [37]. When treated with digoxin, this drug mainly relieves the symptoms of congenital heart disease complicated by acute heart failure by positive inotropic action and negative frequency effect, because the myocardial cells and myocardial contractility are damaged obviously [38]. As a result, the negative frequency mechanism of digoxin is limited, so the improvement of cardiac function of some children is not good [39, 40]. Besides, digoxin can increase the oxygen consumption of cardiomyocytes and lead to digitalis poisoning [41]. In comparison, milrinone can repair cardiomyocyte injury through both positive myodynamia and vasodilation [42–44]. With the continuation of milrinone treatment, the effects of milrinone on cardiac output and myocardial contractility can effectively improve their cardiac function [45, 46].

Some limitations of this study must be considered. Firstly, no foreign RCTs on this issue were found, and the sample size of the study included in this meta-analysis was small, there were differences in treatment plans, which may have a certain impact on the evaluation results. Secondly, the included studies did not explicitly report the blind method application, and did not mention the possibility of hidden distribution and bias, which may affect the credibility of our results. Thirdly, the differences in evaluation indicators of each study increased the heterogeneity of our study. More RCTs with high quality and large samples from different areas and populations are needed in the future, to further investigate the effects and safety of milrinone in the treatment of severe pneumonia and heart failure in children with CHD.

## Conclusions

Routine treatment combined with milrinone in the treatment of heart failure caused by severe pneumonia in children with CHD can quickly improve the symptoms and signs, and promote the recovery of cardiac function with low incidence of adverse reactions. However, considering the limitations of this study, it is necessary to design larger, multicenter RCTs in the future to evaluate the efficacy of milrinone in the treatment of heart failure caused by severe pneumonia in children with CHD.

## Data Availability

All data generated or analyzed during this study are included in this published article.

## Abbreviations

CHD: Congenital heart disease
CNKI: China national knowledge infrastructure
RCTs: Randomized controlled trials
PRISMA: Preferred Reporting Items for Systematic reviews and Meta-Analyses.
LVEF: Left ventricular ejection fraction
MD: Mean difference
95%CI: 95% confidence interval
RR: Relative risk
